# Study on the mechanism of production of γ-PGA and nattokinase in *Bacillus subtilis* natto based on RNA-seq analysis

**DOI:** 10.1186/s12934-021-01570-x

**Published:** 2021-04-09

**Authors:** Min Li, Zilong Zhang, Shenwei Li, Zhengan Tian, Xia Ma

**Affiliations:** 1 School of Perfume and Aroma Technology, Shanghai Institute of Technology, Shanghai, 201418 China; 2 State Key Laboratory of Dairy Biotechnology, Shanghai Engineering Research Center of Dairy Biotechnology, Dairy Research Institute, Bright Dairy and Food Co., Ltd, Shanghai, 200436 China; 3 Shanghai International Travel Healthcare Center, Shanghai Customs District P. R, Shanghai, 200335 China

**Keywords:** *Bacillus subtilis* natto, γ-PGA, Nattokinase, RNA-seq, Co-production mechanism

## Abstract

Poly-γ-glutamic acid (γ-PGA) and nattokinase (NK) are the main substances produced by *Bacillus subtilis* natto in solid-state fermentation and have wide application prospects. We found that our strains had higher activity of nattokinase when soybeans were used as substrate to increase the yield of γ-PGA. Commercial production of γ-PGA and nattokinase requires an understanding of the mechanism of co-production. Here, we obtained the maximum γ-PGA yield (358.5 g/kg, w/w) and highest activity of NK during fermentation and analyzed the transcriptome of *Bacillus subtilis* natto during co-production of γ-PGA and NK. By comparing changes in expression of genes encoding key enzymes and the metabolic pathways associated with the products in genetic engineering, the mechanism of co-production of γ-PGA and nattokinase can be summarized based on RNA-seq analysis. This study firstly provides new insights into the mechanism of co-production of γ-PGA and nattokinase by *Bacillus subtilis* natto and reveals potential molecular targets to promote the co-production of γ-PGA and nattokinase.

## Introduction

Poly-γ-glutamic acid is a natural multifunctional biopolymer composed of D- and/or L-glutamic acid units linked by γ-amide bonds [1]. The molecular weight is in the range of 10–1000 kDa and is synthesized mainly by microbial fermentation [2, 3]. With the predominant characteristics such as non-toxic, biocompatibility and biodegradable [1]. γ-PGA has the functions of thickening, gelation, emulsification, film formation, moisturizing, adhesion, etc. [4]. It is being gradually applied in the fields of cosmetics, food processing, agriculture, medicine, environmental protection and other fields [5–8].

Nattokinase is a biological enzyme extracted from fermented soybeans [9]. It is a linear chain of amino acid chains with spatial folding, produced in the form of a signal peptide, a propeptide and a mature peptide, with a molecular weight of 27.7 KDa [10]. The catalyticternary system of the NK enzyme is Ser-His-Asp [11]. Its characteristic substrate is fibrin and its thrombolytic effect is much stronger than that of snake venom fibrinolytic enzyme, urokinase (UK) and lumbrokinase. The fibrinolytic activity of NK is 4 times higher than that of plasmin and its molecular weight is much smaller than that of urokinase and lumbrokinase. In addition, it is well absorbed by the intestine [12–14]. Therefore, nattokinase is currently considered as a potential drug for the preventing and treatment of cardiovascular diseases [15, 16].

*Bacillus subtilis* natto is able to produce both γ-PGA and nattokinase efficiently [17, 18]. In *B. subtilis* natto, γ-PGA is encoded by the synthetic genes *pgsB*, *pgsC*, *pgsA* and *pgsE* [19, 20]. In contrast, nattokinase is synthesized by the 1143 bp gene aprN, initially called as subtilisin NAT [21]. It would be of great economic benefit if γ-PGA and NK could be co-produced simultaneously by *B. subtilis* natto and both could reach the level of single fermentation. γ-PGA is an important component of natto that promote nutrient utilization. It maintains the moisture in the solid medium and stimulates the production of nattokinase under solid state fermentation conditions [22]. However, many studies have been conducted on the simultaneous fermentation of γ-PGA and nattokinase, less attention has been given to the simultaneous fermentation of γ-PGA and NK by *B. subtilis* natto. Liquid-state fermentation has been successfully applied in co-production strategies to produce high value-added bioproducts [23]. Solid-state fermentation is used for the production of γ-PGA and nattokinase. For example, *Bacillus subtilis* GXA-28 produced γ-PGA and fibrinolytic enzyme by solid-state fermentation [24]. Another successful synergistic production strategy is the simultaneous production of γ-PGA and nattokinase by solid-state fermentation using *B. subtilis* natto [25].

In this study, we investigated the co-production mechanism of γ-PGA and nattokinase by RNA-seq and explored the fermentation process and differentially expressed genes in: 6 h (NK production time), 9 h (γ-PGA production time) and 24 h (NK maximum activity time). The effects of *Bacillus subtilis* natto used in the co-production of γ-PGA and nattokinase were systematically investigated. Then, the up-regulated and down-regulated genes were analyzed to identify the key ones. Combined with the analysis of main metabolic pathways such as carbohydrate metabolism, the potential target genes for enhancing nattokinase activity and γ-PGA yield were observed. To our knowledge, this is the first report to reveal the mechanism of co-production of γ-PGA and nattokinase. The results of this study will help us to better understand the mechanism of production of γ-PGA and nattokinase by solid state fermentation and lay the foundation for the transformation of high-yielding strains with γ-PGA and nattokinase.

## Materials and methods

### Microorganisms, culture media and culture conditions

*Bacillus subtilis* natto was used throughout the experimental study and was cultivated in the following medium: 20 g soybeans, 1.5 g saccharose, 0.4 g glutamate, 0. 025 g MgSO_4_·7H_2_O, 0.025 g K_2_HPO_4_·3H_2_O, 0. 05 g calcium chloride. The seed culture (5%, v/v) was transferred into 50 mL of the fermentation medium in a 250 mL flasks. The strain was statically cultured at 37 °C for 24 h. The cultures were diluted to 5 × 10^5^ cells/mL and harvested to prepare total RNA. The soybeans were washed and soaked overnight in water at 24 ℃. Then the soaking water was discarded. Twenty grams of soaked soybeans were placed in 100 mL gauze conical flasks sterilized at 121 ℃ for 20 min and inoculated with 1 × 10^5^ CFU/g of *B. subtilis* natto. The fermentation process was carried out at 37 ℃ for 36 h in oxygen limitation conditions (cap tighten). All experiments were performed independently in triplicate and the reported results represent the averages of three replicate experiments.

### Determination of γ-PGA and nattokinase

The cells in the fermentation broth were harvested by centrifugation (4 ℃, 8000×*g* for 20 min) and washed three times with PBS (pH 7.0). The concentration of γ-PGA was determined by photometric method with cetyltrimethylammonium bromide (CTAB) assay [26–28]. γ-PGA formed water-insoluble complexes with CTAB, which increased the turbidity of the solution. Samples were taken from shaking flasks and centrifuged (16,000×*g*, 10 min, 4 ℃). The turbidity at 400 nm was measured for quantification of γ-PGA in the supernatant. For this purpose, 100 µL 0.7 M CTAB (2% NaOH) was added to 100 µL sample or standard solution in a 96-well microtitration plate [29]. Incubation turbidity was measured in a Synergy MX microplate reader after 3 min.

The activity of nattokinase was measured by Tos‐Arg‐OMe (TAME) method. TAME can be cleaved by NK to Tos‐Arg and CH_3_OH. CH_3_OH is then oxidized by potassium permanganate to CH_2_O and the chromotropic acid reacts with it to form a blue-violet compound, which has a sensitive UV–visible absorption peak at 574 nm [30]. Briefly, 0.1 mL of TAME solution (0.1 mol L^−1^), 0.1 mL of phosphate buffer (pH 8.0) and 0.1 mL of enzyme solution were mixed in color comparison tubes and kept in a water bath (37 °C) for 30 min. Then, 0.2 mL of TCA (15%) was added to terminate the reaction and 0.1 mL of KMnO_4_ (2%) was added into the tubes and shaken for 2 min. NaSO_4_ (0.1 mL, 10%) was added to reduce excess KMnO_4_. Chromotropic acid (0.4%, 4 mL) was added to terminate the reaction by keeping the mixture in boiling water bath for 25 min, followed by ice-water bath for 10 min. Samples were obtained and its UV‐vis absorbance spectrum was recorded at 574 nm using a Perkin‐Elmer Lambda UV‐vis spectrophotometer.

### RNA isolation and RNA-seq

Total RNA was extracted from 3 samples of bacteria up to 6th, 9 th and 24 th h using the RNA pre-purification Cell/Bacteria Kit (Tiangen Biotech Co, Ltd, Beijing, China), and quality checked using the 2100 Bioanalyzer [31]. Qualified RNA samples were digested with 10U DNaseI (Takara, Japan) at 37 °C for 30 min. Ribo-Zero™ Magnetic Kit (Gram-Negative Bacteria or Gram-positive Bacteria) (Epicentre, USA) was used to remove rRNA after DNase digestion of RNA. After adding Ribo-Zero Reaction Buffer and Ribo-Zero rRNA Removal Solution (Gram-Negative Bacteria or Gram-positive Bacteria) were added, the volume was fixed to 40 μL and the reaction was carried out at 68 °C for 10 min. The sample was then placed at room temperature for 5 min. The processed RNA was added to the pre-washed magnetic beads, mixed thoroughly immediately, placed at room temperature for 5 min and then reacted at 50 ℃ for 5 min, immediately placed on a magnetic stand for more than 1 min. The supernatant was sucked and water was added to 180 μL. 3 M Sodium Acetate, Glycogen (10 mg/mL) and 600 μL of absolute ethanol were added and placed at—20 ℃ for more than 1 h. The Solution was centrifuged to obtain a precipitate,and dissolved in water to form rRNA-depleted RNA.

### RNA-Seq data analysis

The RNA library construction of samples at three time points (6 th, 9 th and 24 th h) was completed by the Shanghai Human Genome Research Center. 2 × 150 bp paired-end sequencing was performed using the Illumina X10 (Illumina, San Diego, CA, USA). Sequence reads for all samples were cleaned using the FASTX toolkit (http://hannonlab.cshl.edu/fastx_toolkit/). After adaptor trimming and quality trimming, the clean reads were mapped to the *B. subtilis* natto genome using Bowtie2 (-very-fast-local) [32]. The reads number of each gene was firstly transformed into RPKM (Reads Per Kilo bases per Million reads) [33], then the MARS model (MA-plot-based method with the random sampling model) in the DEGseq package [34] was used to calculate the gene expression difference between each two samples. We defined simply genes with at least twofold change between two samples and FDR (false discovery rate) less than 0.001 as differential expressed genes.

Up-regulated or down-regulated genes of *B. subtilis* natto in culture was filtered using FDR ≤ 0.05, fold change ≥ 2, or FDR ≤ 0.05, fold change ≤ -2. The filtered genes were associated with the Gene-ontology (GO) terms and the Kyoto Encyclopedia of Genes and Genomes (KEGG) functions. Fischer' s exact test was used and restricting analysis to functional groups with more than 2 genes. Enrichment score greater than or equal to 1 for GO term and pvalue less than 0.05 for KEGG functions were set as the stringency. For KEGG, the color of the element appearance was set by FDR ≤ 0.05, fold change ≥ 2, or FDR ≤ 0.05, fold change ≥ − 2.

## Results and discussion

### Co-producing of γ-PGA and Nattokinase

In this study, the growth curves of the viable cells, the γ-PGA yield and nattokinase activity curve were obtained under these conditions. The analysis of the growth curves in soybean culture medium revealed that the logarithmic growth period occurred mainly between 6 and 24 h. CFU decreased slightly when γ-PGA was produced at 9 th h and then growth resumed slowly around 24 th h, but the process was slow. When the growth reached to 36 th h, the biomass could basically reached the same level as the 16 th h. Therefore, the period of 24–36 h was the growth recovery period and the change of biomass from 36 to 40 h was negligible. The number of viable cells at 6 th, 9 th and 24 th h were 1.2 × 10^5^, 1.6 × 10^6^, 1.0 × 10^8^ CFU/g, respectively.

**Fig. 1:**
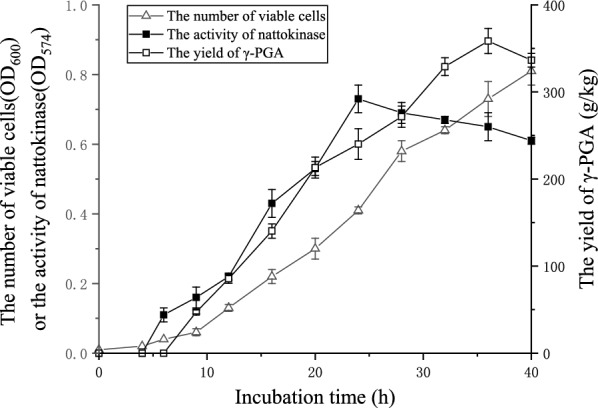
Time courses of the number of viable cells, the activity of nattokinase and the yield of γ-PGA under solid-state fermentation in 250 ml flasks

In the previous work, *B. subtilis* natto could produce high-yields of γ-PGA and nattokinase under the solid-state fermentation, respectively [35, 36]. Based on this phenomenon, a communal study of γ-PGA and nattokinase was conducted in this study using soybeans as substrate. The addition of carbon sources and sodium glutamate to the substrate significantly increased the production of γ-PGA and nattokinase [24, 37, 38]. Among them, sucrose was chosen as the carbon source based on the previous studies that glucose, sucrose and fructose have similar effects on γ-PGA production [39]. Therefore, *B. subtilis* natto was cultured in sucrose and sodium glutamate medium at 37 °C to monitor the growth during fermentation (Fig. 1). After continuous monitoring of fermentation for 40 h, nattokinase production was found at 6 th h and γ-PGA production at 9 th h.

**Fig. 2:**
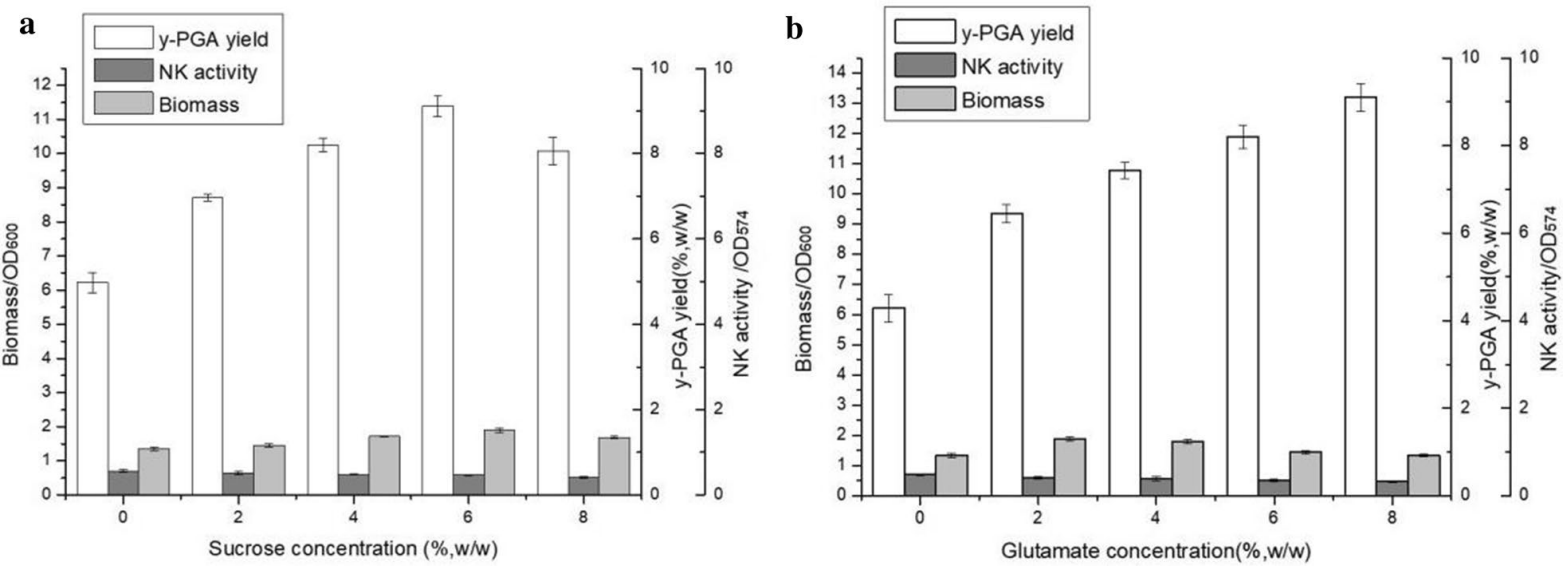
Effect of different sucrose concentration (**a**) and glutamate concentration (**b**) on γ-PGA yield, NK activity and biomass of *B. subtilis* natto

As shown in Fig. 1, the maximum γ-PGA yield (358.5 g/kg, w/w) was obtained when 50 g sucrose was added per kilogram of substrate. The number of viable cells increased rapidly after 4 h, reaching the highest value (5.13 × 10^9^ CFU/g) at 24 th h and remained stable thereafter. It indicates that the present co-production conditions have no adverse effect on the growth of bacteria. Interestingly, the time course of γ-PGA yield and nattokinase activity were synchronized, increasing rapidly between 6 and 36 h before reaching the maximum yield of γ-PGA (358.5 g/kg, w/w) and maximum activity of nattokinase (1388 U/g). Then, the activity of nattokinase remained stable constant after 24 h, but the concentration of γ-PGA decreased significantly after 36 h. This may be caused by γ-PGA depolymerase, which is heavily excreted by *B. subtilis* natto in the late stationary phase [40]. This indicates that both γ-PGA and nattokinase can be produced with the available medium components. Taking into account of the γ-PGA yield, NK value and extraneous nutrient cost, 5% (w/w) sucrose, 4% (w/w) glutamate and 37 ℃ were selected for further studies (Fig. 2).

By analyzing the change of γ-PGA concentration and nattokinase activity at different periods, it was found that the synthesis and accumulation of γ-PGA occurred mainly in the logarithmic growth phase, which is consistent with the study of Mahaboob Ali [41]. The maximum number of viable cells was reached at 36 h with a stable period from 12 to 20 h and a declining period from 20 to 40 h. From the relative activity curve of NK, it was found that nattokinase was gradually synthesized as the bacteria grew, which is consistent with Gao Z' s research [42]. With the increase of γ-PGA concentration, the viscosity of the fermentation broth will gradually increase, which makes RNA extraction difficult [43]. Therefore, the transcription of *B. subtilis* natto was analyzed at 6 th, 9 th and 24 th h in this study.

### Analysis of transcriptome of *B. subtilis* natto

**Fig. 3:**
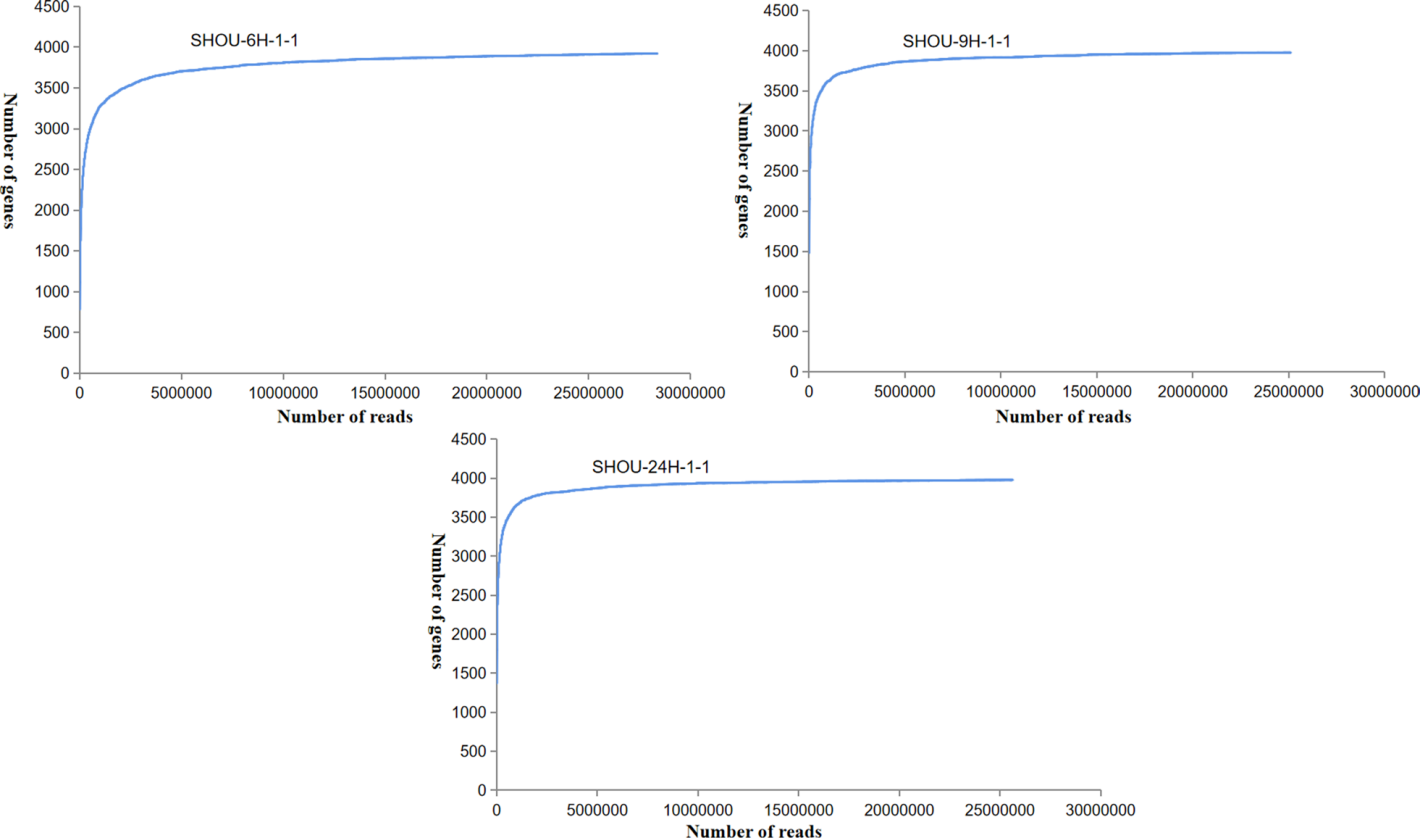
Saturation analysis of sequencing for 6 th, 9 th, 24 th h: *B. subtilis* natto: by RNA-seq

It was found that the adjustment of sucrose and sodium glutamate content in the medium did not affect the production time of γ-PGA and NK, which might be related to the expression of related genes during the fermentation. Therefore, in order to systematically analyze the interaction between γ-PGA and nattokinase co-production process, the gene expression of *B. subtilis* natto at different fermentation periods was investigated using RNA-seq. More than 30 million high-quality base pairs reads were generated in each samples. In addition, saturation analysis showed that sequencing depth was saturated (Fig. 3).

The data of three stages of *B. subtilis* natto were analyzed separately. More than 3800 protein-coding genes were expressed during the fermentation process, accounting for 99.19% of the total 4023 genes. Significantly expressed genes were obtained mainly by DEGseq software analysis (Fig. 7). Specific pathways in differentially expressed genes were grouped based on the KEGG database. The proportion of genes involved in carbohydrate metabolism was up-regulated, including the PTS system and pyruvate metabolism, etc. The proportion of up-regulated genes involved in carbohydrate metabolism, including the PTS system and pyruvate metabolism, was increased. Many of these functionally related genes were up-regulated, which indicated that these pathways are activated under co-production conditions to provide sufficient substrate and energy to maintain cell survival (Table 1).

**Table 1:** Important genes exhibiting changed expression in different times

Gene name	Gene annotation	6th h RPKM	9th h RPKM	24th h RPKM
*AprE*	Subtilisin AprE	53.89	1677.84	26090.62
*glnA, GLUL*	Glutamine Synthetase	1313.58	5606.32	308.68
*gudB, rocG*	Glutamate Dehydrogenase	1.95	25.45	49.05
*pgsB*	Poly-gamma-glutamate synthase PgsB	25.62	1462.01	47.47
*pdhC*	Pyruvate dehydrogenase E2 component	3.43	23.92	2681.45
*pdhD*	Dihydrolipoamide dehydrogenase	8.11	25.41	2760.24
*gltD*	Glutamate synthase small subunit	100.37	1465.77	99.54
*gutB*	Glutamate synthase large subunit	122.12	1179.40	104.15
*aspB*	Aspartate aminotransferase	732.16	1620.20	216.93

**Fig. 4:**
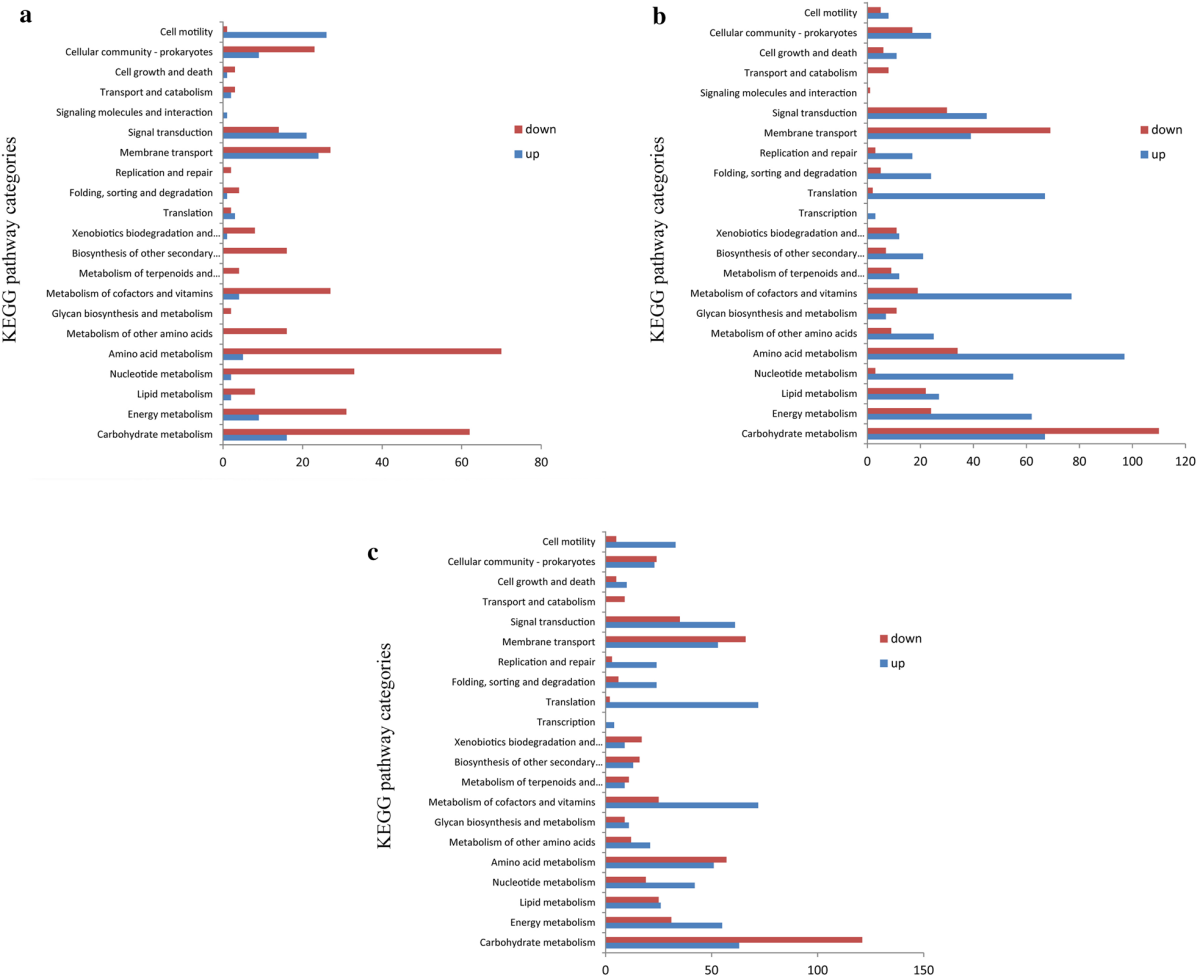
MA plots for differentially expressed genes. Significantly up and down differentially expressed genes are highlighted in red and blue dots, respectively

There were significant differences in the expression of genes related to amino acid synthesis, especially those related to glutamate, aspartic acid, histidine and serine metabolism at 9 th h and 24 th h compared to 6 h. Glutamate-oxoglutarate amidotransferase (GOGAT) and glutamate dehydrogenase (GDH) are essential for the synthesis of glutamate [44]. In this study, the gene *glnA* encoding GOGAT was up-regulated. However, the two genes encoding GDH (*gud B* and *roc G)* showed opposite transcriptional changes. The changes in the transcription levels of the genes indicated that glutamine synthesis is not inhibited by nattokinase (Fig. 4).

Under the co-production environment, γ-PGA production is directly related to the up-regulation of glutamate synthesis- related genes and the down-regulation of γ-PGA degrading enzymes. It is reported that the genes encoding these proteins can also be expressed in *Bacillus licheniformis* under salt stress conditions, which provides a direction for screening of industrial strains of *Bacillus subtilis* [37]. We found that the three *ycg MNO* genes were up-regulated in the co-production environment, which is consistent with the results in *Bacillus subtilis* [38]. This might be the reason for the co-production of γ-PGA and NK simultaneously during fermentation. Based on the analysis of the above results, the yield of γ-PGA and the activity of nattokinase may be improved in *B. subtilis* natto through several metabolic patterns.

**Fig. 5:**
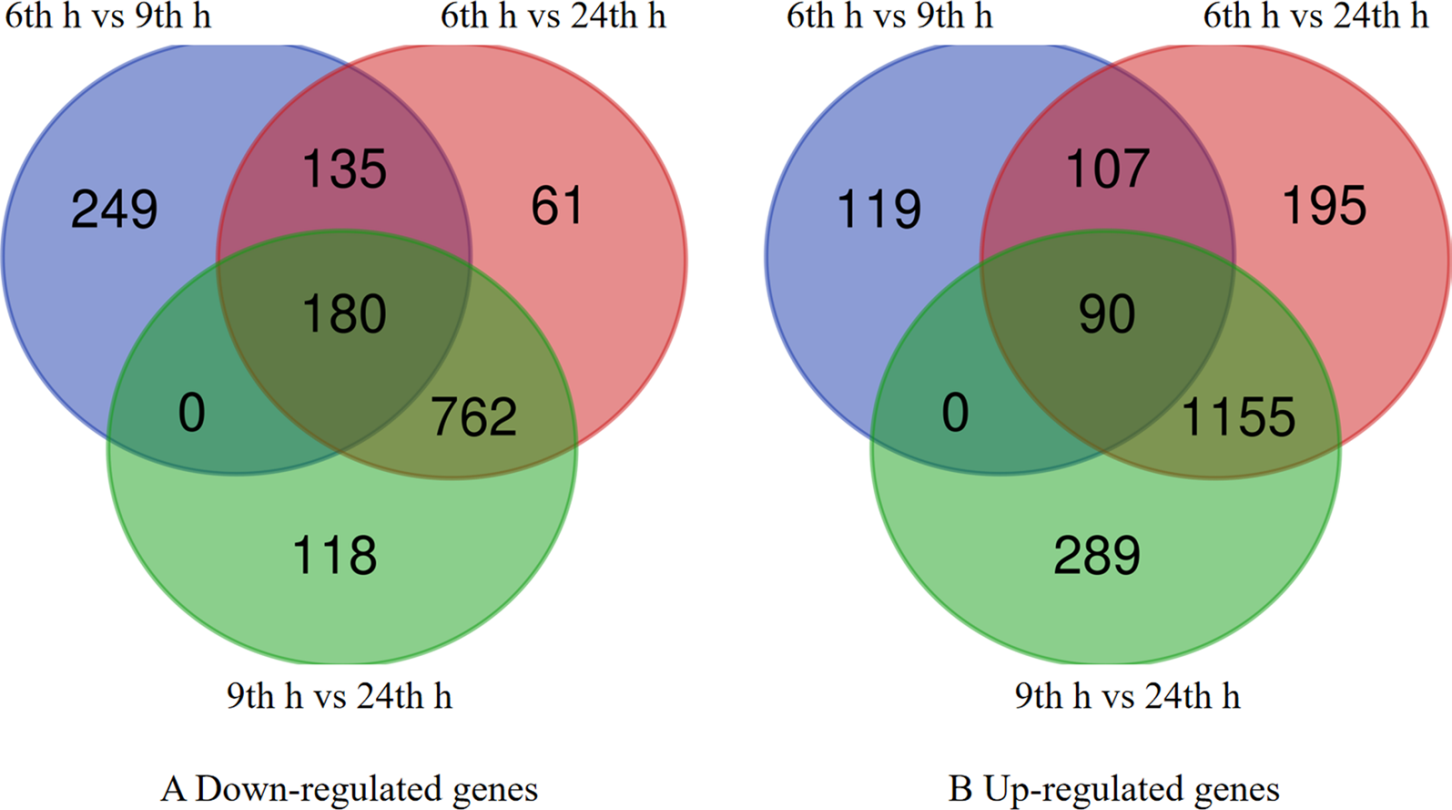
Venn diagrams of the number of differentially expressed genes in the three pair-wise comparisons

To analyze the regulation of *B. subtilis* natto, we focused on three pair-wise comparisons. As can be seen in Fig. 5, 249 genes were up-regulated between 6 and 9 h, and 119 genes were down-regulated between 6 and 9 h. Significantly different genes for amino acid metabolism were down-regulated between 6 and 9 h, probably because the rate of NK synthesis was affected by γ-PGA synthesis [45]. Significantly different genes for amino acid metabolism were up-regulated from 9th h to 24 th h, which was consistent with the highest activity of nattokinase at 24 h during fermentation. 118 genes were up-regulated between 9 and 24 h, and 289 genes were down-regulated between 9 th h and 24 th h. It may be related to the inhibition of the expression of the synthetic genes of γ-PGA when the synthesis rate of nattokinase is high.

**Fig. 6:**
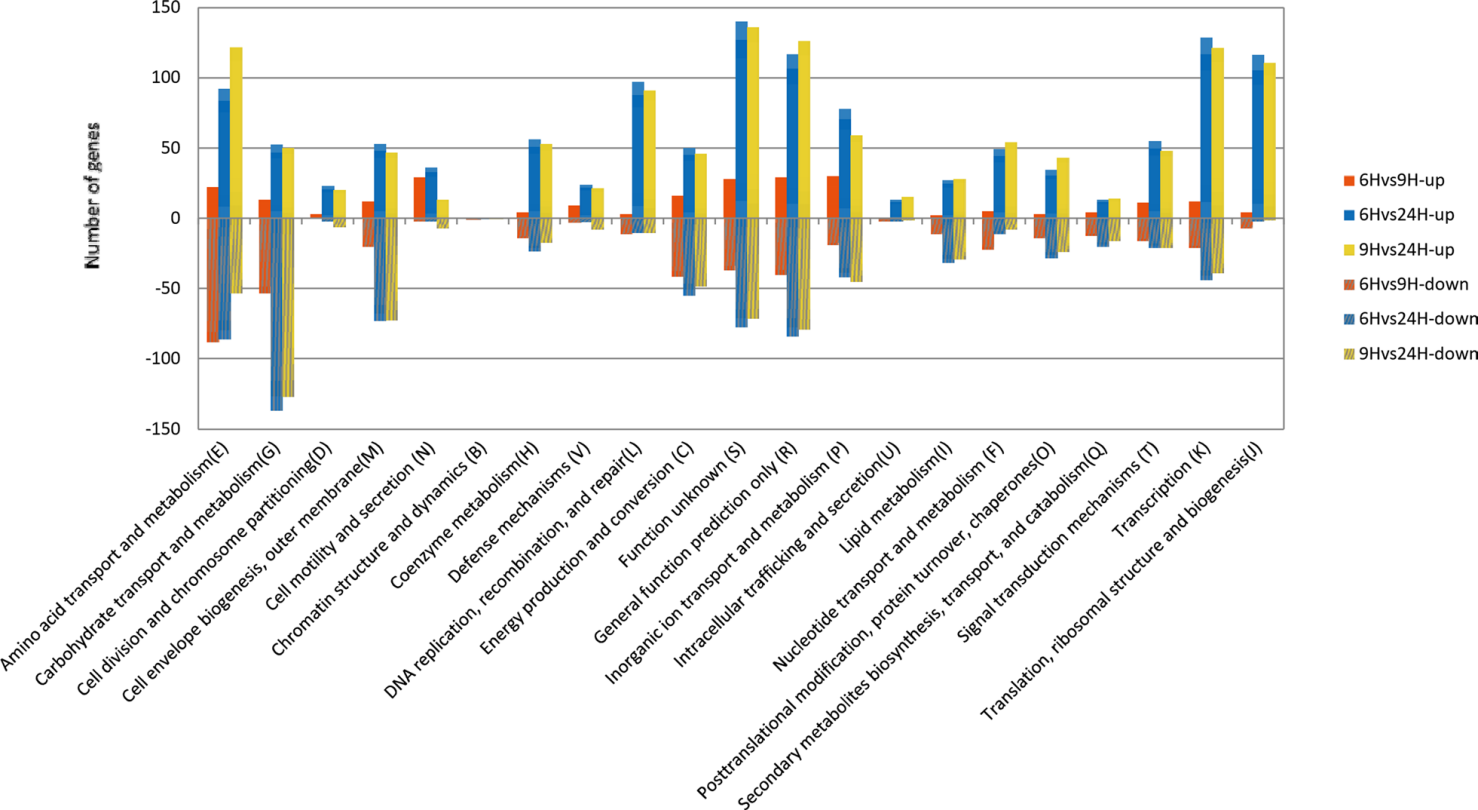
Cluster of Orthologous Groups (COG) functional categories of genes induced and repressed under different conditions. Each bar represents the number of DEGs in each category in the *B. subtilis* natto genome. Orange bars indicate genes repressed at 9 th h relative to 6 th h; The diagonal orange bars indicate 9th h significantly up-regulated genes relative 6 th h. Blue bars indicate genes repressed at 24 th h relative to 6 th h and the diagonal Blue bars indicate 9 th h significantly up-regulated genes relative 6 th h. Yellow bars indicate genes repressed at 24 th h relative to 9 th h and the diagonal yellow bars indicate 24 th h significantly up-regulated genes relative 9 th h

**Fig. 7:**
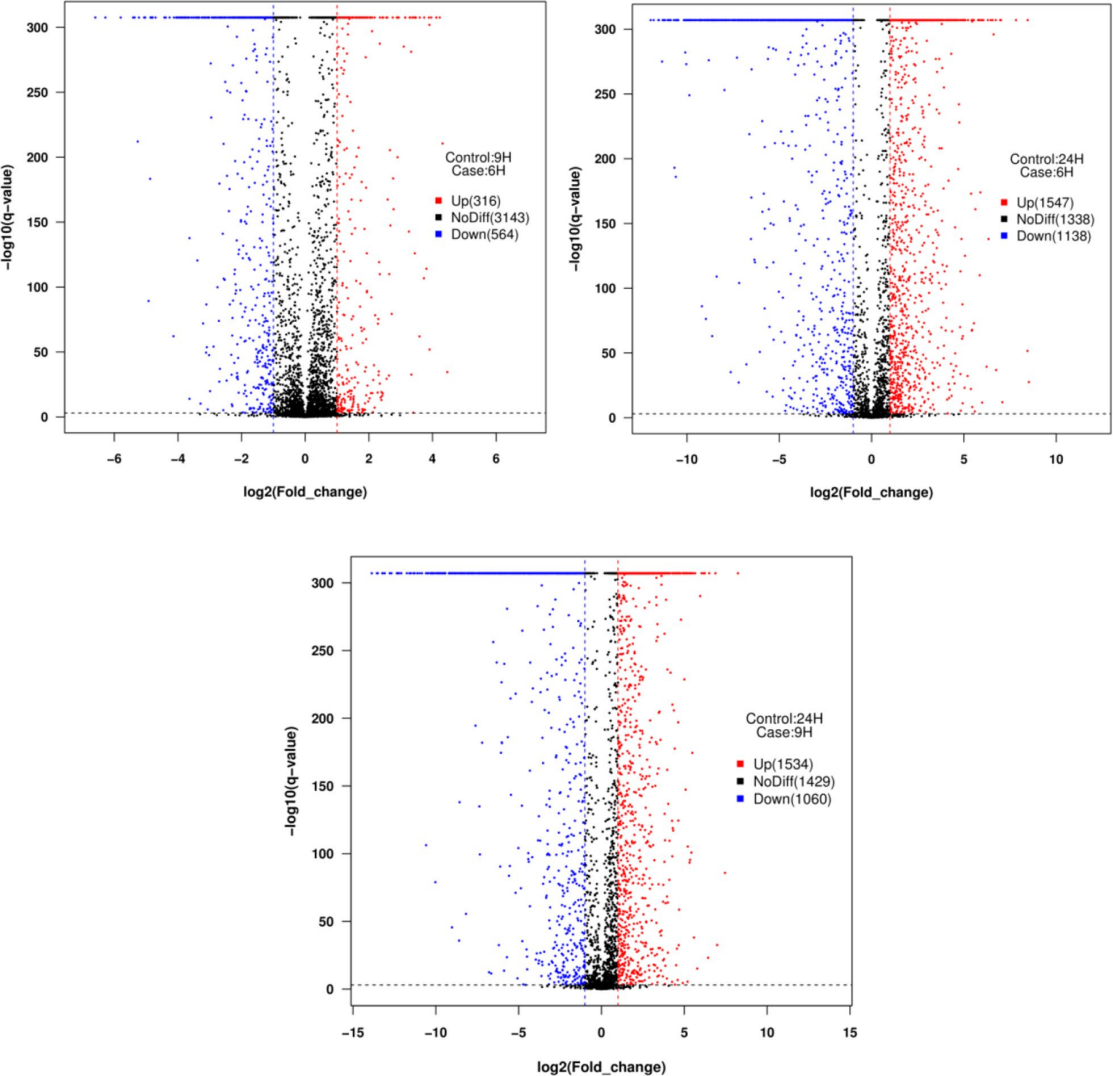
The distribution of up-regulated and down-regulated genes was compared by three pairs of KEGG pathway categories. **a** The strains of *B. subtilis* natto grown on soybean medium were compared with 6 th and 9 th h. **b** The strain of *B. subtilis* natto grown on soybean medium was compared with 9 th and 24 th h. **c** The strain of *B. subtilis* natto grown on soybean medium were compared with 6 th and 24 th h

In order to investigate the functional information of these DEGs, the genes were classified according to COG function (Fig. 6). The figure shows that most of the functional classes contain both up-regulated and down-regulated genes, suggesting that *B. subtilis* natto needs to balance metabolic pathways in soybean culture to maintain cell survival. In general, more genes were up-regulated than down-regulated under co-production conditions. Many metabolic pathways were significantly affected by the co-production conditions, such as amino acid metabolism, energy metabolism and carbohydrate metabolism. Many functionally related genes are up-regulated, which indicates that these pathways are activated to provide γ-PGA and nattokinase under co-production conditions (Fig. 7).

**Fig. 8:**
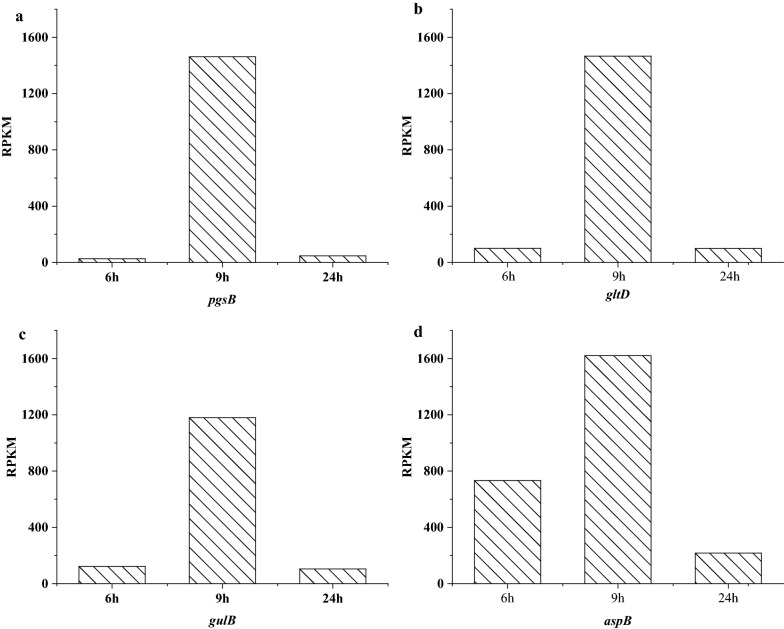
The expression level (RPKM) of *pgs B* (poly-gamma-glutamate synthase *Pgs B*), *glt D* (glutamate synthase small subunit), *gul B* (glutamate synthase large subunit) and *asp B* (Aspartate aminotransferase) at 6 th, 9 th and 24 th h were used to measure expression in *B. subtilis* natto

When *B. subtilis* natto was cultivated in soybean medium, the expression level of genes related to energy and carbon metabolism was greatly affected by co-production (Fig. 4). Among of the 124 energy and carbon metabolism-related genes, 100 genes were significantly down-regulated and 24 genes were significantly up-regulated when 9 th h vs. 6 th h. In addition, the expression of energy conversion-related genes such as BSNT_RS01180 (NAD(P)H-quinone oxidoreductase) and BSNT_RS20605 (cytochrome bd ubiquinol oxidase) were also affected to express. Synthesis-related genes encoding γ-PGA and NK should be up-regulated and expressed separately to improve the ability of cells to produce these two substances. However, the *pgs B* and *aprN* genes were not up-regulated in *B. subtilis* natto in response to the co-production environment. The result is consistent with *Corynebacterium glutamicum* and *Bacillus licheniformis* [46, 47]. Although this result is inconsistent with our expectations, this response to co-production seems to be similar in all three strains (Fig. 8).

**Fig. 9:**
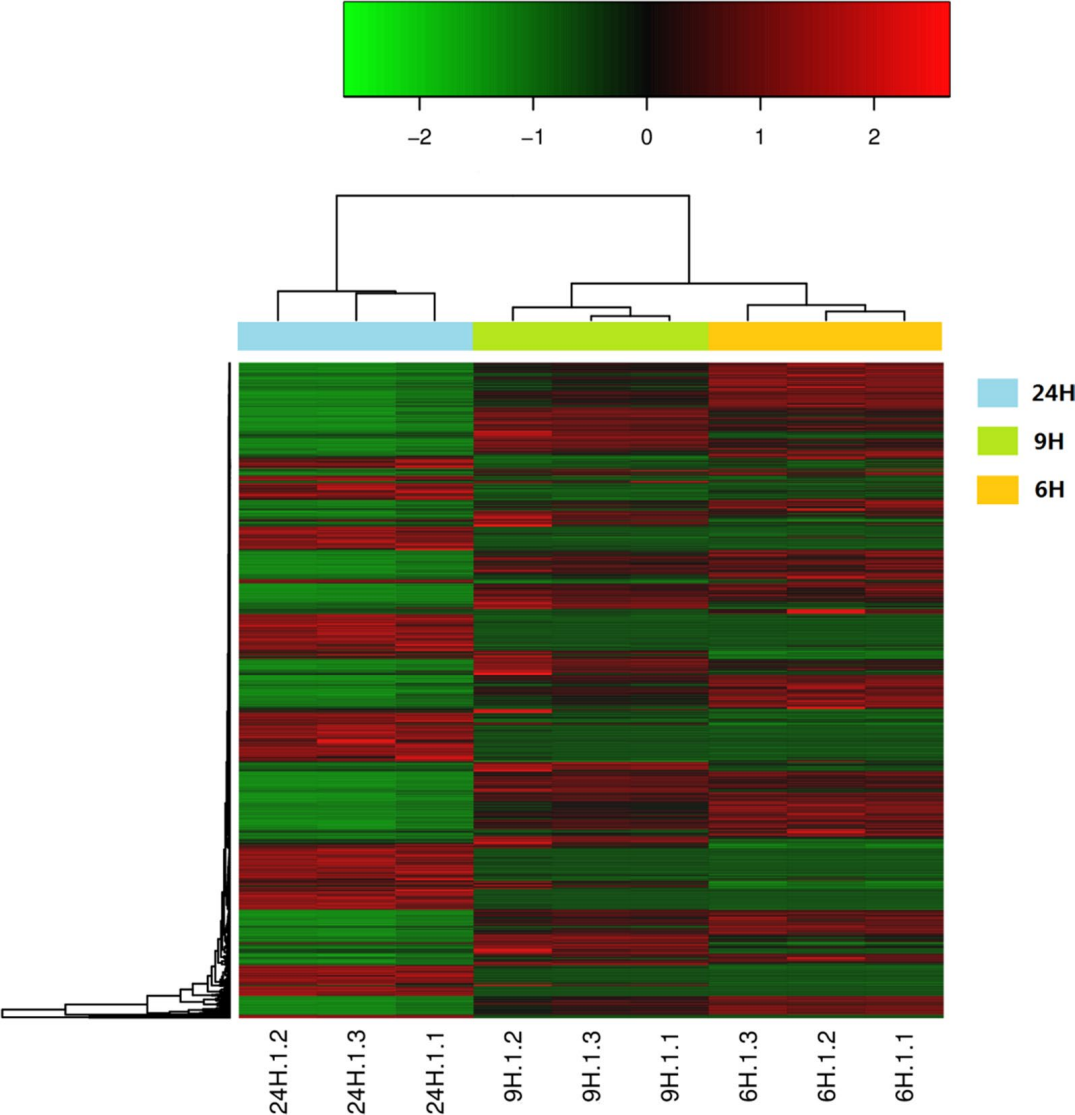
Differentially expressed genes of *B. subtilis* natto under solid-state fermentation, in duplicated experiments, which show consistency in regulation of gene expression. Each horizontal line represent the expression level of a gene. Red and green indicate up- and down-regulated genes, respectively. In the culture, the most transcripts of *B. subtilis* natto were down-regulated. (For interpretation of the references to color in this figure legend, the reader is referred to the web version of this article.)

The production of γ-PGA requires the involvement of glutamate dehydrogenase (GDH). Experiments showed that the gene expression level of glutamate dehydrogenase was increased significantly from 6 to 9 h, which is consistent with the previous reports [48]. The expression level of *pgs B* was continuously increased, which is consistent with the synthesis of γ-PGA [49]. However, the expression level of glutamate transferase was gradually decreased at three time points, suggesting that the incorporation of endogenous glutamate synthesis in addition to γ-PGA synthesis. This phenomenon is associated with glutamate-dependent strains that require the addition of large amount of exogenous glutamate, while only 10% of endogenous glutamate is synthesized [5]. The expression of *apr N* was gradually increased at three time points, demonstrating that NK was not restricted by co-production conditions during fermentation. At the same time, a large number of amino acids were used in the cellular synthesis of nattokinase and the pentose phosphate pathway is one of the key ones [50] (Fig. 9).

During the co-production process, the synthesis of γ-PGA and nattokinase is affected by the synthesis pathways of substances other than these two as by-products. Polysaccharides are one of the by-products of soybean fermentation. Not only the production of γ-PGA and nattokinase is affected, but also the viscosity of the fermentation broth and the metabolism of bacteria. As seen from the Fig. 6, the gene expression of polysaccharide biosynthetic protein began to decrease after 9 h with the production of γ-PGA. It could be proved that the production of polysaccharides is not a key factor affecting the viscosity of the fermentation broth in the co-production process.

### Identification of key modules for co-production of γ-PGA and nattokinase

**Fig. 10:**
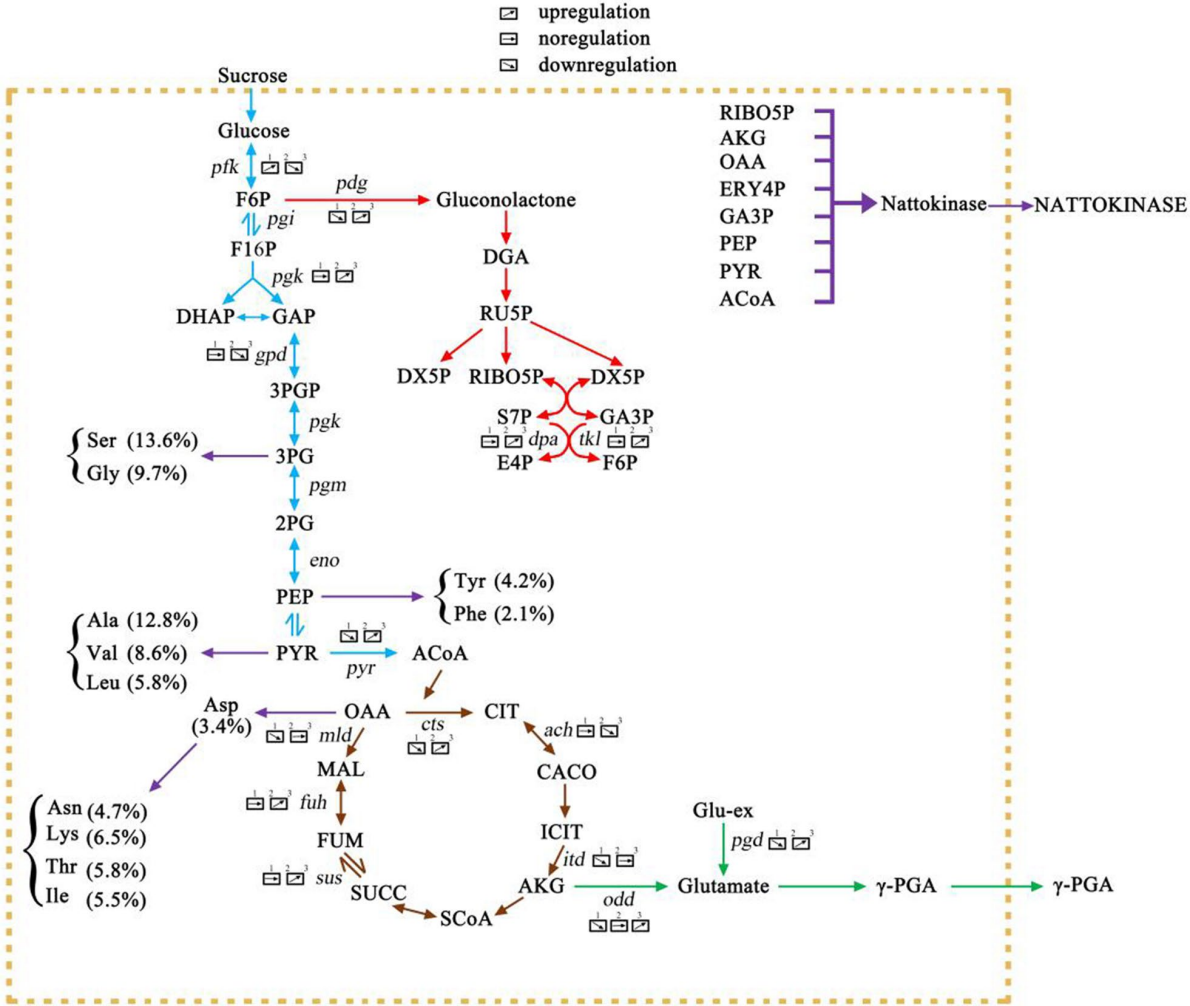
Overview of key genes and related pathways in transcription analysis. 1. *B. subtilis* natto was compared with 9 h at 6 h; 2. *B. subtilis* natto was compared with 24 h at 9 h; 3. *B. subtilis* natto was compared to 24 h at 6 h. Gene annotations downloaded from NCBI: Glucose, glucose; G6P, glucose 6-phosphate; F6P, fructose 6-phosphate; F16P, fructose 1,6-diphosphate; GA3P, glyceraldehyde 3-phosphate; DHAP, phosphate Dihydroxyacetone; 3PGP, 1,3-diphosphoglycerate; 3PG, 3-phosphoglycerate; PEP phosphoenolpyruvate; PYR, pyruvate; ACoA, acetyl-CoA; CIT, citric acid; OAA, grass Acylacetic acid; MAL, malic acid; FUM, fumaric acid; SUCC, succinic acid, SCoA, succinyl coenzyme A; AKG, α-ketoglutarate; ICIT, isocitrate; CACO, cis-aconitic acid; Glutamate, gluten Glycine; γ-PGA, γ-polyglutamic acid; Glu-ex, exogenous glutamic acid; Gluconolactone, gluconolactone hexaphosphate; DGA, 6-phosphogluconate; RU5P, 5-phosphate ribulose; DX5P, xylulose 5-phosphate; RIBO5P, ribose 5-phosphate; S7P, sedoheptulose 7-phosphate; E4P, erythrose 4-phosphate

The differentially expressed genes involved in central metabolism are shown in Fig. 10. When *B. subtilis* natto was cultivated in soybean medium, most of the genes related to Carbohydrate cycle, Amino acid synthesis and the γ-PGA synthesis were upregulated, whereas genes related to glutamate degradation were downregulated.

Compared with the gene expression level at 6 h, the gene expression levels of glucose dehydrogenase, acetate dehydrogenase, γ-PGA synthase and isosulfate synthase in glycolysis pathway at 9 h were down-regulated. The expression levels of genes such as acid dehydrogenase were all down-regulated. Acetyl-CoA is completely oxidized through the TCA cycle and synthesizes intermediates required for ATP production and other synthetic pathways such as amino acid biosynthesis [51]. Glutamic acid is the precursor for the synthesis of γ-PGA and is mainly generated from α-ketoglutarate from the TCA cycle [52]. This means that γ-PGA produced at 9 th h is almost supplied by exogenous glutamic acid. The glucose 6-phosphate dehydrogenase gene is down-regulated, indicating a slightly inhibition of the PPP metabolic pathway by γ-PGA synthesis during nattokinase synthesis. Comparing the gene expression levels at 24 th h and 9 th h, glyceraldehyde triphosphate dehydrogenase and pyruvate dehydrogenase were down-regulated. During this period, the gene expression levels of glutamate dehydrogenase and γ-PGA synthase were up-regulated, demonstrating that the synthesis of γ-PGA in Fig. 7 was not affected by nattokinase. It indicates that the highest yield of γ-PGA is affected due to the systematic enhancement of glutamic acid synthesis. In recent years, researchers have proposed to improve the supply of glutamate in cells is improved through several strategies to enhance the production of γ-PGA, which is suitable for a wide range of compounds produced through glutamate-intensive pathways [5]. In the pentose phosphate pathway, the synthesis of nattokinase may be affected by the synthesis of polysaccharides. During fermentation, polysaccharide synthesis genes were down-regulated, which proved that the synthesis of polysaccharide was inhibited from the synthesis of nattokinase and γ-PGA. This is advantageous for the synthesis of γ-PGA and nattokinase. In contrast, the transcript levels of genes involved in the glucose transport and ammonium transport pathways were not significantly different in the present study, which might be a relatively little effect on the glutamate dependence in *B. subtilis* natto [53].

Combined with the fermentation process, it can be seen that the activity of NK was stable after 24 h, while the γ-PGA reached, the highest yield of at 36 h. Subsequently, a part of the γ-PGA was degraded due to the action of γ-PGA depolymerase and γ-PGA was mainly excreted in the fixation stage by *B. subtilis* natto [24, 38].

### Analysis of pentose phosphate pathway (PPP), tricarboxylic acid cycle (TCA) and glycolysis metabolism

Metabolomics analysis of strains containing newly generated promoter sequences of different intensities revealed the key metabolites most associated with the synthesis of γ-PGA and nattokinase. The metabolites from central carbon metabolism are ribose-5-phosphate (R5P), pyruvate and dihydroxyacetone phosphate (DHAP), as well as acetyl-CoA gene expression [47]. The homeostasis of these metabolites can be explained by their prominent function in carbon and nitrogen metabolism [54]. Serine and R5P are two metabolites directly derived from glycolysis intermediates glycerol 3-phosphate and glucose-6-phosphate (G6P). Therefore, the down-regulation of serine and R5P synthesis genes can be explained by a greater demand for the TCA cycle. The supply of precursors produced by γ-PGA is limited by the activity of TCA. The results show that citrate synthase, aconitase and malate dehydrogenase form a protein complex that catalyzes the sequential reaction of the TCA cycle, supplying the synthesis of γ-PGA and nattokinase. In addition, the synthetic genes of 2-oxoglutarate dehydrogenase complex and glutamate synthase are affected by these protein–protein interactions. NADPH is a cofactor for glutamate synthesis. Therefore, the higher demand for glutamate also leads to a higher demand for NAD(P)H in the co-production and fermentation of γ-PGA and nattokinase. Here, the gene expression levels of NAD^+^ and NADP are also significant.

In addition, PEP is the metabolite most associated with γ-PGA and NK synthesis. With the increase of γ-PGA production rate, the expression level of PEP gene was up-regulated. In addition to PEP, further intermediates of central carbon metabolism also were improved with the yield of γ-PGA [55]. Glutamine is a metabolite that is negatively related to the higher rate of γ-PGA synthesis. It is a substrate for glucose re-synthesis to glutamate and its concentration is closely related to the amount of glutamate required. Proline and NADP^+^ concentrations are affected by the increased demand of glutamate for higher γ-PGA production. Succinic acid is another metabolite and its demand for glutamate has changed because the carbon flux at the branch point of 2-oxoglutarate can point to glutamate or succinate. Changes in the succinic acid synthesis genes show a weak relationship between succinate concentration and γ-PGA yield. A comparison of 2-oxoglutarate, succinic acid and glutamic acid concentrations (Fig. 10) shows this connection.

## Discussion

Studies have shown that the high-yield mechanism of γ-PGA is complicated in co-production fermentation, involving the supply of glutamate, the transport and secretion of γ-PGA, energy metabolism, glutamate metabolism, etc. The biosynthesis and regulation of γ-PGA and the biosynthesis and regulation of other complex related pathways may be regulated by various regulatory factors, which is a very complex system. Therefore, the transcriptome of this strain was compared by RNA-seq under co-production conditions. The RNA-seq library was constructed from samples at three time points: 6 th h (production of nattokinase); 9 th h (production of γ-PGA); 24 th h (the highest activity of nattokinase). For example, *glt ABC* (co-coding glutamate-α-ketoglutarate amidotransferase), *glt P* (glutamate transporter) and *ycg MNO*, etc. were all up-regulated, while *gud B* (coding glutamate dehydrogenase) was down-regulated. Changes in the expression levels of these genes promote the increase of glutamate production, resulting in a higher yield of γ-PGA in the co-production environment. The synthesis genes of γ-PGA are *pgs B, pgs C, pgs AA* and *pgs E*, among which *pgs B* and *pgs C* are responsible for the synthesis of γ-PGA, *pgs AA* and *pgs E* are responsible for transporting the synthesized γ-PGA outside the cell [56]. A gene, *pgd S*, encodes γ-D /L glutamyl hydrolase, which promotes the release of γ-PGA. The transcription of tricarboxylic acid cycle (TCA)-related enzymes from α-ketoglutarate to oxaloacetate is inhibited [55, 57] and the transcription level of α-ketoglutarate to proline synthase is significantly increased, indicating that *B. subtilis* natto has a proline response co-production mechanism. In addition, the synthesis of TCA-related enzymes from α-ketoglutarate to oxaloacetate was inhibited, thus ensuring the synthesis of proline from a large amount of α-ketoglutarate.

During fermentation, Glu severs as the starting molecule leading to the synthesis of Asp and Gln via the reactions mediated by aspartate aminotransferase and glutamine synthetase, respectively. Therefore, when Asp or Gln is directly supplied from the medium without Glu, a moderately high levels of glutamate synthase are present in response the the elevated Glu pool in *B. subtilis* natto, thereby resulting in an increase of Glu. Taken together, it indicates that Glu plays a determining role in the synthesis of nattokinase [50]. The highest NK-yielding pathways catabolize glycerol into ATP and NADPH using glycolysis and the pentose phosphate pathway (PPP) and produce CO_2_ as by-product [58, 59]. The synthesis pathways of NK use the incomplete tricarboxylic acid (TCA) cycle and do not produce any fermentative by-products. The reverse reaction between PEP and pyruvate and two anaplerotic reactions are inactive for NK synthesis. However, these reactions are activated when biomass is produced (Fig. 1). The synthesis of nattokinase requires the oxidative branch and non-oxidative branch of PPP as appropriate precursors for amino acid production, while the production of biomass does not utilize the oxidative branch of PPP [60]. These results indicate that cell growth and the synthesis of enzymes and γ-PGA follow different paths and do not interfere with each other.

## Conclusion

In conclusion, we found that *B. subtilis* natto can produce nattokinase at the same time without affecting the production of γ-PGA in the solid-state fermentation. We use the fermentation method of Japanese natto to produce γ-PGA in a targeted manner and nattokinase while minimizing by-product output. As a result, the maximum activity of nattokinase (1388 U/g) was obtained at 24 h and the highest yield of γ-PGA(358.5 g/kg) was obtained at 36 h. In this study, our results provide the first insights into the co-production mechanism of γ-PGA and NK of *B. subtilis* natto in the solid-state fermentation. Genes involved in carbohydrate metabolism, amino acid metabolism and γ-PGA synthesis pathways were investigated by RNA-seq that was used to explore the potential factors and metabolic pathways for the synthesis of γ-PGA and nattokinase and initially summarized the production mechanism of the co-production of γ-PGA and nattokinase. It could guide the molecular improvement of the genome of *B. subtilis* natto, the propagation of *B. subtilis* natto with high-efficiency and large-scale production of target metabolites.

## Data Availability

Not applicable.
